# Genetic analysis of roots and shoots in rice seedling by association mapping

**DOI:** 10.1007/s13258-018-0741-x

**Published:** 2018-09-21

**Authors:** Yan Zhao, Cong-hui Jiang, Rashid Muhammad Abdul Rehman, Hong-liang Zhang, Jinjie Li, Zi-chao Li

**Affiliations:** 1 Key Lab of Crop Heterosis and Utilization of Ministry of Education and Beijing Key Lab of Crop Genetic Improvement, China Agricultural University, Beijing, 100193 China; 2 Central Hi-Tech Lab, University of Agriculture Faisalabad, Sub-campus Burewala, Vehari, 61010 Pakistan

**Keywords:** Rice (*Oryza sativa* L.), Root, Shoot, Association mapping

## Abstract

The vigorous shoots and roots help to improve drought resistance and post-transplanting recovery in rice seedlings (*Oryza sativa* L.). Hundreds of loci related to root system have been identified recently, but little research has been done on shoot traits, and the relationship between roots and shoots development is also still unclear. The objective of this study was to identify associated loci for roots and shoots in rice seedlings as well as to screen pleiotropic QTLs involved in coordinated development of roots and shoots. Using mini core collection of 273 cultivated rice accessions and 280 simple-sequence repeat markers, we investigated six traits [root length (RL), root thickness (RT), root weight (RW), shoot length (SL), shoot weight (SW) and ratio of root-to-shoot mass] in seedlings. Study was performed in hydroponic medium and genetic analysis was performed by association mapping using general linear model (GLM) with population structure (Q) and mixed linear model (MLM) involving Q and familial relatedness (K). Two subgroups *indica* and *japonica* showed significant differences in RT, RW and SW. Maximum correlation was observed between RW and SW. Using GLM 65 QTLs for root and 43 QTLs associated with shoot traits were detected. Among them, seven QTLs were present between RL and RW and five common QTLs were detected between SL and SW with high phenotypic variation effects (PVEs). Two key pleiotropic QTLs were also identified involved in collaborative development of roots and shoots in rice seedlings. Importantly, 17 and 10 QTLs were identified for root and shoot traits respectively in both studies of GLM and MLM. More common QTLs with high PVEs between root and shoot traits suggested that longitudinal growth (RL and SL) played an important role in accumulation of biomass (RW and SW). Considering the obvious phenotypic differences and fewer common QTLs between *indica* and *japonica*, we suggested that there could be different mechanisms of seedling development between both subpopulations. Key pleiotropic QTLs and QTLs identified for root and shoot traits in both studies of GLM and MLM could be preferentially used in marker-assisted breeding for strong rice seedling.

**Electronic supplementary material:**

The online version of this article (10.1007/s13258-018-0741-x) contains supplementary material, which is available to authorized users.

## Introduction

The vigor of root system is essential for uptake of water and nutrients, structural support of the canopy and environmental sensing in rice (*Oryza sativa* L.) and other higher plants (Coudert et al. 2010; Orman-Ligeza et al. 2013). Strong shoots are requirement of higher energy utilization efficiency and more photosynthates. Moreover, plants can adjust ratio of root-to-shoot mass (R/S) for different soils conditions and drought stress conditions (Rich and Watt 2013; Xu et al. 2015). Due to global water shortage and physical damage in rice transplanting, strong roots and shoots are conductive to rice growth in middle to late periods and stable yield. Thus research on the underlying genetic mechanisms and discoveries of genes related to root and shoot traits in rice seedling will be helpful for developing elite cultivars with high survivability and tolerance to abiotic stress.

For rice root system, root length (RL), root thickness (RT) and root weight (RW) are important traits required for best survival under severe conditions, such as water or nutrient deficiency. However, the measurements of these traits were considered the most difficult (Li et al. 2005; Price et al. 2002). Up to now, many root traits QTLs have been identified in rice by bi-parental QTL mapping (Xu et al. 2004; Yue et al. 2006; Zhang et al. 2001a, b). A few “hot spots” of 29 root traits (including RL and RT) were detected by meta-QTL analysis using 675 QTLs database of 24 published papers (Courtois et al. 2009). The *qRL6.1* for RL was narrowed in 336 kb region on chromosome 6 under hydroponic culture conditions (Obara et al. 2010). In rice bi-parental mapping population, Bala × Azucena, 83 QTLs were identified for root system architecture by using a semi-automated 3D in vivo imaging and digital phenotyping system (Topp et al. 2013). In 2013, another RL QTL, *qRL7* was mapped to a 657.35 kb region on chromosome 7 using BC_3_F_3_ recombinant lines (Wang et al. 2013). The QTL *qRL7* significantly improved the RL at the heading stage. *DRO1* was the first reported gene associated with deep rooting trait, which could improve the ability of drought avoidance (Uga et al. 2013a, b). Many candidate genes were identified to be related to the development of roots and shoots, by using association mapping of 22,000 SNPs and 180 rice varieties from tropical country Vietnam under greenhouse conditions (Phung et al. 2016). Comparatively, the studies of shoot length (SL) and shoot weight (SW) were relatively neglected as strong shoot traits, due to more research on analogous plant height and yield. Traditional bi-parental mapping was conducive to explore useful QTL with low allele frequency in nature, and provided genetic information to further explore molecule basis of roots and shoots development in rice. Nevertheless, it was hard to ignore that there was only a couple of parental lines utilized in one bi-parental mapping, and the extent of natural variation that can be explored was still limited. In contrast, association mapping could greatly increase the range of natural variation and the number of significant loci (Huang et al. 2010, 2012).

We performed association mapping to explore QTLs for six rice seedling traits, by using 273 varieties of the mini core collection (MCC) of cultivated rice and 280 simple-sequence repeat (SSR) markers. Along with correlations among six rice seedling traits in our population, we further screened pleiotropic QTLs associated with more traits of roots and shoots in *indica* and *japonica*, respectively. The research provided a comprehensive insight into genetic architecture and collaborative development of roots and shoots in rice seedling.

## Materials and methods

### Materials

The 273 varieties were selected from MCC of cultivated rice in our laboratory (Zhang et al. 2011). Among them, 154 and 119 cultivars belong to *indica* and *japonica*, respectively. The plants were grown in hydroponic culture system in Beijing. Seeds were washed with distilled-water and germinated at 32 °C for 64 h. Five plants for each variety with uniform size and better growth potential were transplanted into plastic foam frames with gauze bottom (30 cm × 42 cm). Each frame contained 13 rows of 10 wells with the capacity to handle 110 plants of 22 varieties and 20 plants of 2 guarding rows at a time. Two frames floated in a plastic box (60 cm × 42 cm × 18 cm) with nutrient solution (Table S1) (Yoshida et al. 1976). The pH and concentration of solution were adjusted twice a day with NaOH and distilled water, and the solution was replaced weekly. Plants were grown in natural conditions in China Agricultural University, Beijing (39°N, 116°E), and were protected with tarpaulins on rainy days. Seedlings of each variety were grown in the periods of 24th May to 15th June and 23rd June to 15th July in 2014 as two replications. The average temperature fluctuations of the day were from 19.91 to 31.61 °C and from 22.50 to 31.89 °C in two periods, respectively.

### Phenotyping

Five plants per variety with exception of guarding plant were evaluated and the mean value was considered as the phenotype of a variety. Each plant was cut at the junction between root and shoot. RL and SL were measured with a ruler, and whole roots and shoots were weighed after washing clean and drying surface water using absorbent paper. The thickness of five roots per plant was measured 1 cm down the length of the roots under the microscope (the mean of five thick roots represents the RT of the plant) (Li et al. 2005). The R/S was computed to evaluate the coordination between growth and development of the roots and shoots.

### Genotyping

The DNA was extracted from the tender leaves using cetyl trimethyl ammonium bromide method (Rogers and Bendich 1989). The selected 273 cultivars were genotyped by 280 SSR markers. The 280 SSR markers were deliberately selected with appropriate distance between markers. The average distance between makers was about 835 kb, varying from 0.06 to 4050 kb; 18% of distances between markers were less than 150 kb, more than 50% were less than 750 kb, and more than 90% were less than 1800 kb (Pan et al. 2015). The standard protocols for PCR, electrophoresis and gel staining were followed (Panaud et al. 1996; Zhang et al. 2009).

### Data analysis

The phenotype analysis for six seedling traits was carried out using IBM SPSS Statistics Version 19. One-way analyses of variance (ANOVA) were separately used to test phenotypic significant difference among genotypes for the full population. Broad-sense heritability (*h*^2^) of each trait in the full population was calculated using the formula: *h*^2^ = ${\delta }_{g}^{2}/({\delta }_{g}^{2}+{\delta }_{e}^{2}),$ where ${\delta }_{g}^{2}$ and ${\delta }_{e}^{2}$ were the estimates of genetic, and error variances derived from the mean square expectations of ANOVA, respectively.

Population structure of this MCC has been analyzed at our laboratory (Zhang et al. 2009), and the 273 rice accessions have been clearly divided into *japonica* and *indica* subpopulations, respectively. The percentages of admixtures from structure results (Q matrix) were used as fixed effects in the models [general linear model (GLM) and mixed linear model (MLM)] to correct the association tests for false positives (Figs. S1, S2, S3). The kinship matrix (K matrix) of the MCC was determined using TASSEL V4.0, and further was used as random effect in MLM (Figs. S4, S5, S6). Association analysis between average data of six seedling traits and SSR markers were performed using GLM and MLM by TASSEL V4.0 (Figs. S7, S8, S9, S10, S11, S12). Here we lowered the standard and analyzed more loci from GLM for fully understanding of all QTL for root and shoots, although there could be some false positive signals in these QTL (Figs. S1, S2, S3, S7, S8, S9). Actually, there were hundreds of QTLs identified on the whole rice genome for root traits (including RL, RT and RW) by linkage mapping, and 33 of 65 QTLs for the 3 root traits were re-identified in our association studies under GLM as mentioned in the following “Results” section. The comparison suggested that most of QTLs under GLM were convinced. Moreover, to improve reliability of QTL for effective utilization in marker assisted breeding, we focused on the QTLs co-identified by association mapping of strict MLM (Q + K) and GLM (Q) (Figs. S4, S5, S6, S10, S11, S12).

Conventional Bonferroni multiple test correction and false discovery rate are strategies for the adjustment of false positive discovery, but both strategies just consider the number of input SNP which apparently does not correlate to the complexity of associated trait. The number of quantitative trait nucleotides sometimes reflects the complexity of associated trait and thus was used to adjust the false discovery (Pan et al. 2015; Zhao et al. 2018). Thus, we used the formula “−log_10_(0.05/effective number of SNPs with a *P*-value less than 0.05),” i.e., the threshold at a significance level of 5% after Bonferroni-adjusted multiple test correction. Markers with *P*-values lower than α′ could be QTLs significantly associated with target trait.

The relative genotypic effect for the *i*th allele of a QTL in the *s*th subpopulations for the corresponding indicator was denoted as ${\text{RGE}_{si}}=({\overline{x}}_{si}-{\overline{x}}_{s})/{\overline{x}}_{s}\times 100\%,$ where *s* represented *indica* or *japonica* subgroup, $\overline{x}_{si}$ denoted the phenotypic average of *i*th allele of a QTL in *s*th subgroup, $\overline{x}_{s}$ was the phenotypic average of *s*th subgroup (Pan et al. 2015). The alleles with significantly high RGE for a certain QTL were designated as positive genotypes while the alleles with significantly lower RGE value were designated as negative genotypes. The positive and negative genotypes were judged by using the one sample T-test.

## Results

### Characterization of roots and shoots in rice seedling

**Fig. 1:**
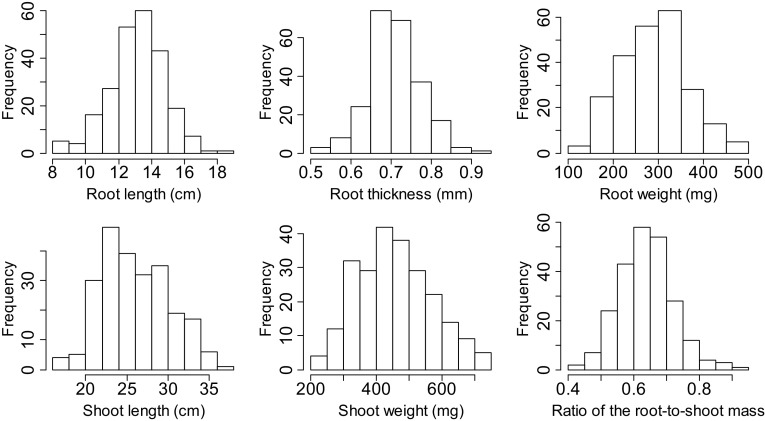
Distribution of six seedling traits in 273 mini core collection of cultivated rice

There were moderate ranges of coefficients of variation from 10.2 to 28.4% for six seedling traits in both replications (Table S2). Because there were slight environmental differences within the optimum temperature range of rice growth, it was appropriate to treat the both datasets from two cultivation periods as two replications. Additionally, the high correlation coefficients between these datasets showed the possibility to treat the periods as the replications in later analysis (Table S2). The ANOVA showed extremely significant differences (*P* < 0.001) among varieties for each trait, which indicated the existence of a large amount of genetic variations in MCC. The values of skewness and kurtosis indicated the continuous and approximately normal distribution of genotypes for all selected traits (Fig. 1; Table S2). The broad-sense heritability was reasonably higher; varying from 66.23% (RT) to 93.32% (SL) in both repetitions, demonstrating that genetic factor played a key role in phenotypic diversity of MCC.

**Fig. 2:**
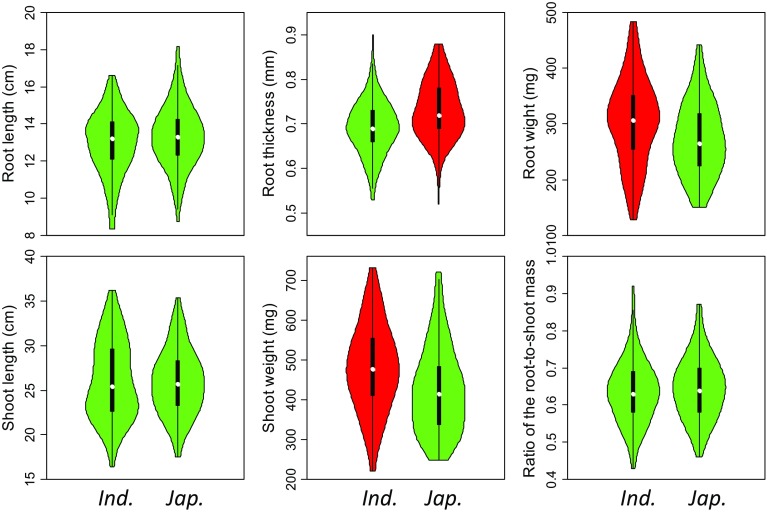
Comparison of six seedling traits between *indica* and *japonica*. The value colored by red is higher than that colored by green with significant difference. Violin plot was a box plot with a rotated kernel density plot on each side, in which white point, black box and black line represented the median of the data, interquartile range and range, respectively

A comparison of root and shoot traits between both subgroups revealed that there were significant difference in SW, RW and RT, except of RL and SL (Fig. 2; Table S3). The roots were thicker in *japonica* than that in *indica*, but the weights of roots and shoots were less in *japonica* than these in *indica*. Extremely significant (*P* < 0.01) positive correlations in both periods were detected between RW with each of SL, RL, SW and R/S, SW with each of SL and RL. Extremely significant negative correlations in two periods were detected between R/S with SL and SW (Table 1). The highest correlation coefficient (0.855) was observed between RW and SW.

**Table 1:** Correlation coefficients of six seedling traits in two periods

Traits	RL	RW	SW	RT	R/S
SL	0.245**	0.425**	0.591**	0.134*	− 0.235**
RL	1	0.514**	0.464**	0.222**	0.156*
RW		1	0.855**	0.221**	0.358**
SW			1	0.145*	− 0.161**
RT				1	0.144*

*Significant correlation at α = 0.05, **significant correlation at α = 0.01

### Genetic architecture of root and shoot traits in rice seedling

**Fig. 3:**
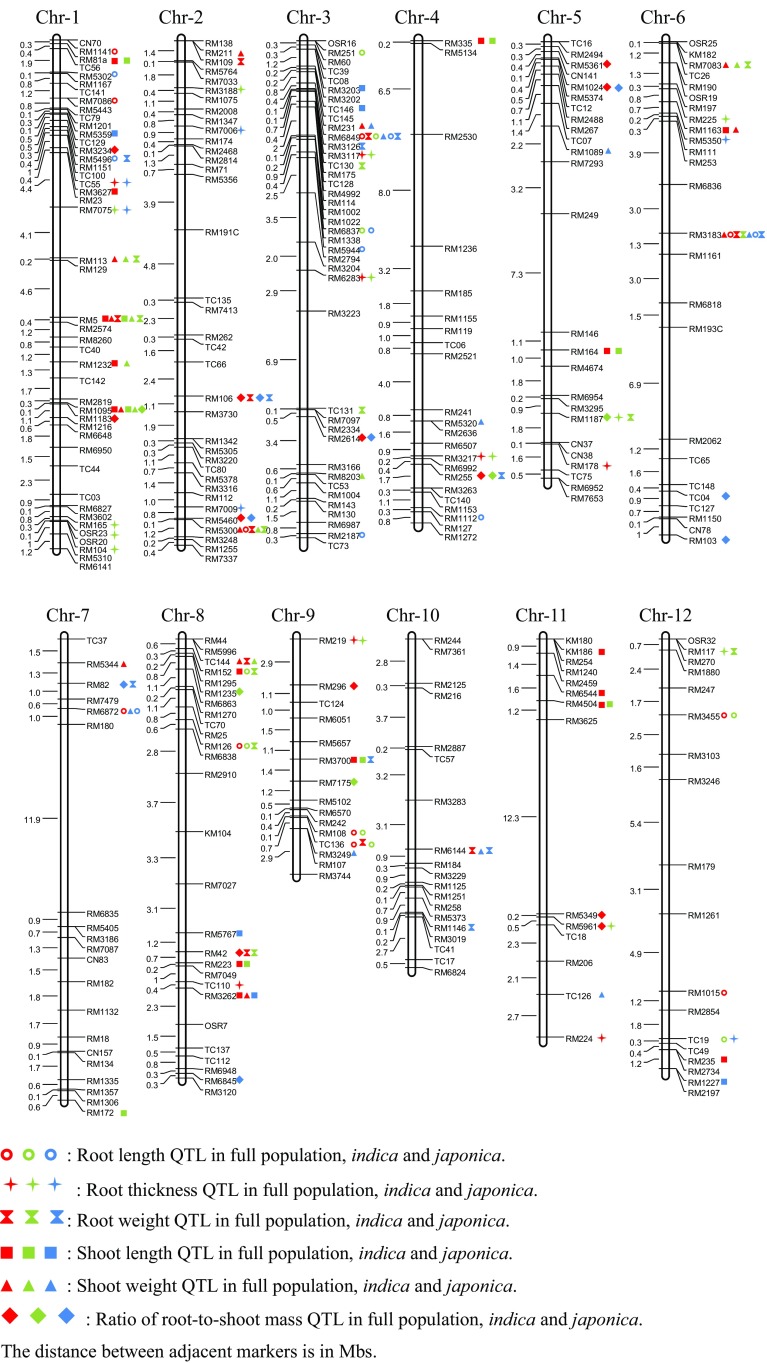
Mapping of QTLs for six traits in rice seedling

Under GLM (Q), 65 QTLs related to root traits in rice seedling were identified. Among them, 20, 21 and 24 QTLs were related to RL, RT and RW respectively (Fig. 3; Table S4), while 7, 16 and 10 QTLs had already been reported for RL, RT and RW in previous studies (Courtois et al. 2009). Two RL governing QTLs (*qRL9-1* and *qRL9-2*) and two RT related QTLs (*qRT2-3* and *qRT4-1*) have repeatedly been reported several times in previous mapping (Courtois et al. 2009). Phenotypic variation effects (PVEs) of QTLs for RL, RT and RW ranged from 6.08 to 21.36, 4.64 to 21.14 and 6.90 to 21.24% respectively (Table S4). We compared the number, types and PVE of QTLs in both subgroups (Fig. 3; Table S4). Two (*qRL3-2* and *qRL3-3*), one (*qRT1-2*) and one (*qRW6-2*) common QTLs were identified in both subgroups for RL, RT and RW, respectively. For RL, 7 of 9 QTLs with total PVE (86.16%) in *indica* could not be detected in *japonica*, and 7 of 9 QTLs with total PVE (117.15%) in *japonica* could not be detected in *indica* as well. The similar trend in RT for 12 of 13 QTLs with high PVE (136.76%) in *indica* and 5 of 6 QTLs with high PVE (79.04%) in *japonica*, and in RW for 11 of 12 QTLs with high PVE (120.42%) in *indica* and 9 of 10 QTLs with high PVE (139.02%) in *japonica* were observed. Additionally, *qRL3-2* and *qRW6-2* with average PVE (9.36 and 13.13%, respectively) were detected in *indica, japonica* and full population which could be inevitable nodes in whole molecular network involved in rice root development.

Only one common QTL was detected to be associated with RL (*qRL12-3*) and RT (*qRT12-2*) with high PVE (9.85 and 13.79%, respectively) (Fig. 3; Table S4). Meanwhile, seven and two RW QTLs were detected to be associated with RL and RT, respectively. The total PVE of seven common QTLs for RL and RW were about 77.10 and 78.45%, and total PVE of two common QTLs for RT and RW were about 20.17 and 21.58%.

Among the total 43 shoot related QTLs identified in this study of GLM (Q), the 22 and 21 QTLs were detected for SL and SW, with PVEs ranged from 6.01 to 28.87 and 5.73 to 22.42%, respectively (Fig. 3; Table S4). Both shoot traits were quantitative but presented lesser PVEs in our population as mentioned above. There were five common QTLs associated with SL and SW with high total PVEs (62.54 and 51.96%, respectively). Due to the highest correlation coefficients of RW and SW in both replications, we identified the common QTLs associated with root traits and shoot traits to explore the mechanism of collaborative development of roots and shoots in rice seedling (Fig. 3; Table S4). Eight SW QTLs (PVE 87.57%) were detected to be associated with RW (85.78%) and four SW QTLs (PVE 44.28%) were detected to be associated with RL (PVE 42.03%). But no common QTL was identified in SW and RT. Meanwhile, three SL QTLs (PVE 43.21%) were detected to be associated with RW (PVE 33.80%) and one SL QTL (PVE 8.29%) was detected to be associated with RL (PVE 13.24%). No common QTL was identified in SL and RT as well. Additionally, among 19 R/S related QTLs, 5 QTLs with total 59.45% PVE were detected to be associated with RW but only 1 QTL with 7.99% PVE was associated with SW.

A total of 30 QTLs for roots and shoots were identified in association mapping of MLM (Q + K) and GLM (Q), including 17 root traits QTLs, 10 shoot traits QTLs and 3 QTLs for R/S (Fig. 3; Table S5). Among 17 root traits QTLs, 1 of 4 RL QTLs, all 6 RT QTLs and 1 of 8 RW QTLs have been identified in previous studies described above. The PVE of QTLs for RL, RT and RW ranged from 7.01 to 18.64, 7 to 21.87 and 6.33 to 32.9%. One important QTL was detected for RT (*qRT5-1*) and RW (*qRW5-1*) with high PVE (14.32 and 12.8%, respectively). Meanwhile, among 10 shoot QTLs detected in association mapping of MLM (Q + K), the 4 and 6 QTLs were explored for SL and SW, with PVEs ranged from 9.85 to 26.87 and 11.89 to 40.61% (Fig. 3; Table S5). Another important QTL was detected for SL (*qSL8-4*) and SW (*qSW8-2*) with high PVE but in different population.

### Pleiotropic QTLs involved in collaborative development of root and shoot in rice seedling

Under GLM (Q), four and three pleiotropic QTLs for RW and SW were detected in *indica* and *japonica*, respectively. The total PVEs of four QTLs related to RW and SW were 46.76 and 55.10% in *indica*, and the total PVEs of three RW and SW QTLs were 52.26 and 39.29% in *japonica*. The seven pleiotropic QTLs could be associated with biomass accumulation of whole rice seedling including roots and shoots, which agreed with high PVE and correlation coefficients between RW and SW (Fig. 3; Tables 1, S4). Under MLM (Q + K), one pleiotropic QTL of GLM (Q) (at marker RM3183 on chromosome 6) was detected to be associated with RW (*qRW6-2*) in full population and *indica* and SW (*qSW6-3*) in *japonica* (Fig. 3; Table S5).

**Fig. 4:**
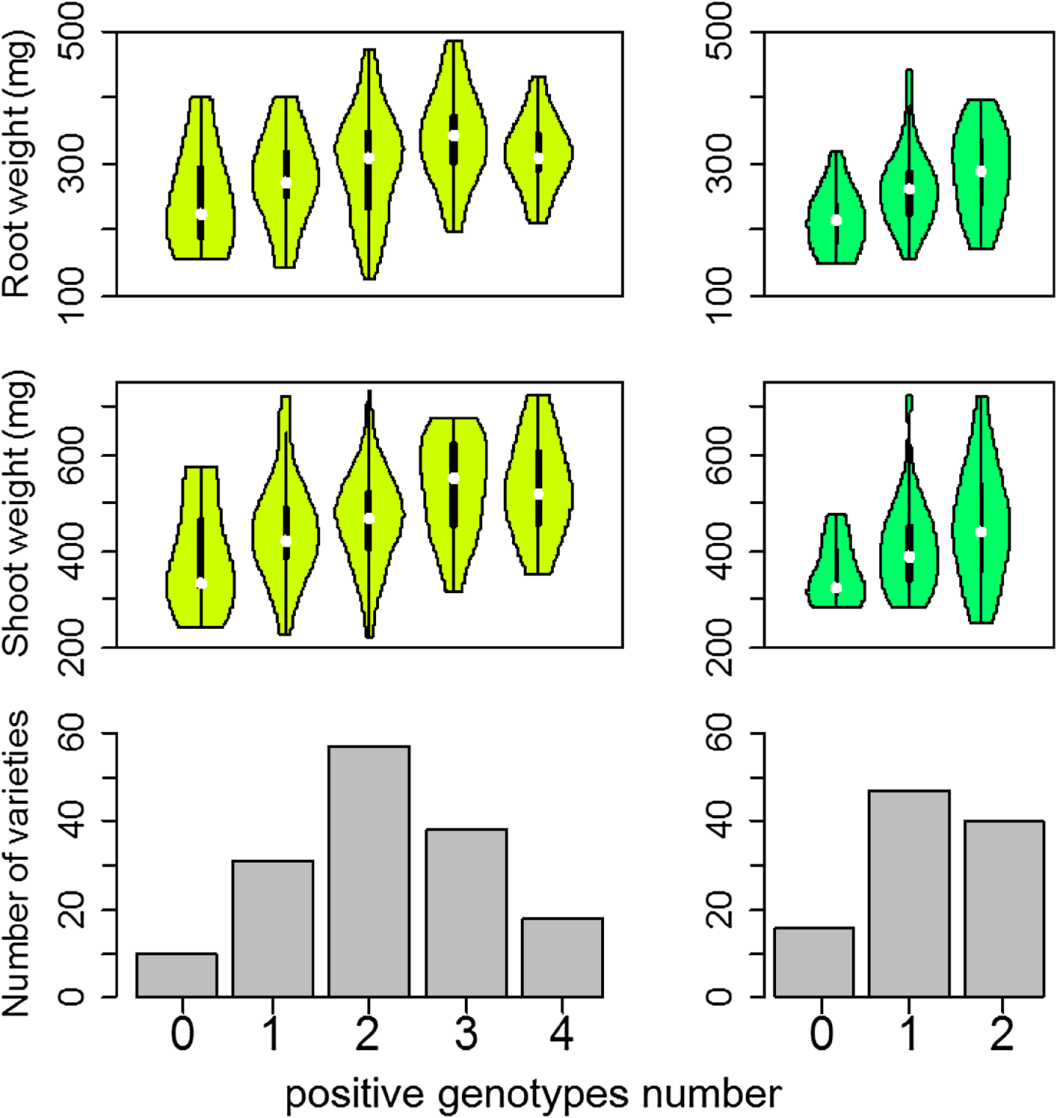
Root weight and shoot weight of rice groups possessing different positive QTLs in *indica* (left) and *japonica* (right). Violin plot was a box plot with a rotated kernel density plot on each side, in which white point, black box and black line represented the median of the data, interquartile range and range, respectively

To provide positive genotypes of the seven QTLs and germplasm resources that may be used in breeding program, we investigated the relative genotypic effect (RGE) of individual genotype for each QTL. Five common positive genotypes and four common negative genotypes of four QTLs for RW and SW were detected in *indica*, which had significant differences to phenotypic average of *indica* (Table 2). And two common positive genotypes and three common negative genotypes of two QTLs (at marker positions RM3183 and RM6144 on chromosome 6 and 10, respectively) for RW and SW were detected in *japonica*, which had significant differences to phenotypic average of *japonica* as well (Table 3). By using these common positive genotypes as selection markers, we independently counted the average RW and SW of varieties containing same positive genotype number in *indica* and *japonica* accessions. The average RW and SW of varieties with the same positive genotype number grew in tandem with positive genotypes number in *indica* and *japonica*, respectively (Fig. 4). Nine of 18 elite varieties with four positive genotypes were screened out as elite varieties in *indica*, given that higher RW (354 mg), SW (592 mg) and more positive genotypes (Table S6). Meanwhile, 20 of 40 elite varieties with two positive genotypes were screened out in *japonica*, due to high RW (340 mg), SW (517 mg) and more positive genotypes as well (Table S7). Additionally, there were higher mean values of RL, RT and SL in the elite varieties (Tables S6, S7).

**Table 2:** Phenotypic effect of four pleiotropic loci in *indica*

Loci	Alleles	Numbers	Root weight		Shoot weight		Shoot length	
			Mean	RGE (%)	Mean	RGE (%)	Mean	RGE (%)
RM113^a^	117:117	102	279	2.81*	448	2.53*	26.47	1.32*
	118:118	25	243	− 8.35*	401	− 6.58*	25.53	− 2.29*
RM5300^a^	102:102	20	316	− 20.17*	496	− 16.54*	24.45	− 6.42*
	104:104	95	295	3.77*	461	3.28*	26.40	1.03*
RM7083^a^	111:111	78	312	− 3*	503	− 3.87*	25.31	− 3.14*
	113:113	53	317	2.66*	518	4.91*	27.17	3.99*
RM5^b^	104:104	18	323	4.1*	520	7.99*	26.85	2.75*
	108:108	26	279	6.3*	430	8.43*	27.75	6.21*
	112:112	39	279	− 8.21*	430	− 10.33*	23.72	− 9.23*

*Significance at 0.05 probability levels

^a^Marker position for pleiotropic QTL associated with root weight (RW) and shoot weight (SW)

^b^Marker position for pleiotropic QTL associated with root weight (RW), shoot weight (SW) and shoot length (SL)

**Table 3:** Phenotypic effects of three pleiotropic loci in *japonica*

Loci	Alleles	Numbers	Root weight		Shoot weight		Root length	
			Mean	RGE (%)	Mean	RGE (%)	Mean	RGE (%)
RM3183^b^	102:102	73	285	4.96*	442	4.09*	13.59	1.86*
	103:103	12	224	− 17.43*	352	− 17.18*	12.33	− 7.6*
	106:106	6	220	− 18.97*	376	− 11.63*	12.72	− 4.68*
RM6144^a^	118:118	47	283	4.18*	446	5.04*	13.22	− 0.93*
	120:120	37	241	− 11.39*	376	− 11.47*	13.09	− 1.92*
RM6849^b^	116:116	16	257	− 5.25*	424	− 0.16	13.52	1.29*
	119:119	76	270	− 0.46	417	− 1.91*	13.21	− 0.99*

*Significance at 0.05 probability levels

^a^Pleiotropic QTL associated with root weight and shoot weight

^b^Pleiotropic QTL associated with root weight, shoot weight and root length

For further evaluation about the relationship among biomass accumulation (RW and SW), RL, RT and SL, we compared the seven pleiotropic QTLs with the QTLs related to other traits. In *indica*, one of four pleiotropic QTLs (at marker position RM5 at chromosome 1) was detected to be involved in SL (21.12%), RW (13.98%) and SW (22.42%) with high PVE enclosed in parentheses. Meanwhile, two of three pleiotropic QTLs (at markers RM3183 and RM6849 at chromosome 6 and 3) were identified to be associated with RL (31.22%), RW (35.87%) and SW (24.13%). Furthermore, in two QTLs (at RM5 and RM3183 position), the RGEs of the genotypes were consistent among three corresponding traits (Tables 2, 3).

## Discussion

To explore the complex genetic architecture of plant development, we measured the six traits related to roots and shoots from 273 seedlings of rice MCC under hydroponic conditions, and performed the association analysis between measured traits and SSR markers in full population, *indica* and *japonica*. Due to the susceptibility of roots to drought, ions and other stimulus (Gamuyao et al. 2012; Henry et al. 2011; Li et al. 2009; Parent et al. 2010), our hydroponics provided the consistent conditions for MCC varieties for repeatable data with high heritability, which was conductive to explore genetic architecture of roots and shoots.

### Characterization of roots and shoots in rice seedling

In various studies it has been observed that there were considerable differences between *indica* and *japonica* in genome and farming conditions (Bridhikitti and Overcamp 2012; Wang et al. 2018). In present study, the results showed that there were differences on developmental orientation between both subgroups, which may due to adaptation of respective farming and water conditions. For *japonica*, thick and long roots and high R/S were conductive to adapt drought conditions in North China. Strong ability of lateral growth and tillering could be the cause of heavier roots and shoots in *indica*, due to that there were similar RL and SL in both subgroups and there were sufficient water conditions for *indica* in South China and Southeast Asia.

The correlation analysis revealed the highest correlation coefficients between RW and SW, which indicated that the development of roots and shoots were interdependent and interactive and the harmony of biomass between roots and shoots could be a key factor of collaborative development of rice plants. Additionally, higher correlation coefficients between RW and RL, SW and SL suggested the longitudinal growth potential played an important role in producing strong seedlings in rice (Table 1).

### Genetic architecture of root and shoot traits in rice seedling

Among three root traits, RW was a complex trait because the accumulation of biomass was determined by a combination of the RL, RT and number of roots. Similarly, SW was determined by SL and number of tillers as well.

In view of the fact that QTLs by bi-parental mapping could be rare mutation with low frequency in natural population, to explore the genetic architecture of roots and shoots in rice seedling, we performed the association mapping by GLM using different populations (full population, *indica* and *japonica*). Including the already known QTLs, a total 65 and 43 QTLs for root and shoot related traits were observed. Among the 65 QTLs observed for root traits 7, 16 and 10 QTLs had already been reported for RL, RT and RW, by using bi-parental mapping in previous studies (Champoux et al. 1995; Courtois et al. 2003; Hemamalini et al. 2000; Horii et al. 2006; Kamoshita et al. 2002; Li et al. 2005; MacMillan et al. 2006; Nguyen et al. 2004; Price et al. 2002; Redoña and Mackill 1996; Xu et al. 2004; Yadav et al. 1997; Yue et al. 2006; Zhang et al. 2001a, b). Our association mapping confirmed the critically important role of known QTLs in cultivated varieties and showed their usefulness in drought breeding with strong roots.

The variable range in PVE for different QTLs showed that root traits were complex quantitative, influenced by a large number of minor genes, which agreed with studies by bi-parental mapping (Courtois et al. 2009; Li et al. 2005, 2009). Given that there were significant differences in RT and RW between *indica* and *japonica* cultivars, and the root traits were indicating the pyramid of several minor genes.

Considering the phenotypic differences and root traits related QTLs between *indica* and *japonica* as mentioned in results, we concluded that *indica* and *japonica* evolved respective mechanisms of development orientation to adapt the different water conditions in rice seedlings.

In this study it was found that the longitudinal growth played an important role in the shoot biomass accumulation in rice seedling as the value of correlation coefficients between RW and RL was significantly higher. It also revealed that the different developmental mechanism for RL and RT, which can influenced the RW together through longitudinal and horizontal growth. Long roots had more effects on RW than thick roots, considered that there were more common QTLs and higher correlation coefficients between RL and RW. However, none of common SL and SW QTLs was identified between *indica* and *japonica*, which suggested the obvious difference between both subgroups in shoot traits as well as root traits.

Taken together, we concluded that long roots were conductive to the accumulation of biomass of roots and shoots but thick roots could not; the longitudinal growth in roots and shoots were interactive, even though there could be different mechanisms involved in root and shoot development. Nevertheless, the root development was proactive in accumulation of biomass of whole rice seedling.

### Pleiotropic QTLs and collaborative development of root and shoot in rice seedling

Exploration of loci and natural elite genotypes underlying root and shoot traits in rice seedling are conducive to improve drought resistance and post-transplanting recovery. In the studies, many QTLs for six root and shoot traits were identified, including several pleiotropic loci. Further evaluation of positive genotypes for these QTLs was performed, and a total of 29 elite varieties with positive genotypes were identified. The availability of pleiotropic loci showed the involvement of complex collaborative development of plant architecture. Further evaluation of individual genotypes for these QTLs showed the average RW and SW of varieties with the same positive genotype number grew in tandem with positive genotypes number in *indica* and *japonica*, respectively (Fig. 4). The results suggested that these QTLs were obvious additive effect loci, which could be used respectively in future rice breeding for strong roots and shoots. Additionally, the screening and selection of elite genotypes with common QTLs showed that the elite varieties and selection markers could be conductive to further research activities of seedling development and strong-seedling breeding.

In our studies, there were low correlation and less common QTLs among RL, RT and SL, which suggested that there were different genes and networks associated with the three traits. However, seven pleiotropic QTLs related to RW and SW with high PVE implied that there were superior genes or networks influenced the collaborative development of roots and shoots, by regulating subordinate genes or networks associated with RL, RT and SL. The two pleiotropic QTLs (at position RM5 and RM3183) could play key role in the network of collaborative development of roots and shoots. Given that the different genotypes of two pleiotropic QTLs influenced several root and shoot traits with consistent positive and negative effect, we considered the two pleiotropic QTLs as node QTLs in collaborative development of roots and shoots. Moreover, the identification of pleiotropic QTLs provided the information about markers that may be used in drought resistance breeding for strong roots and shoots, even though the mechanism of collaborative development of roots and shoots was unclear. It was also observable that the two subgroups did not share the same set of collaborative development mechanism of roots and shoots, due to water condition for *indica* and drought condition for *japonica*.

Long and thick roots and high R/S could be typical characters of *japonica* as a drought-resistance subgroup, basing on comparison of phenotypes and QTLs between *indica* and *japonica* mentioned above. There were upland *japonica* in our MCC with highest RL (14.49 cm), RT (0.78 mm) and R/S (0.66), which could be the useful materials for drought-resistance breeding with extreme phenotypes of RL, RT and R/S. Furthermore, there was higher biomass accumulation (RW and SW) in *indica*. Because of little differences in RL and SL between *indica* and *japonica*, and thinner root in *indica*, we suggested the stronger tillering capacity in *indica* than that in *japonica*. The features of *indica* and *japonica* were formed gradually during rice cultivation and the identification of these features was conductive to comprehensive utilization of advantages of both subgroups in future rice breeding.

## Electronic supplementary material

Below is the link to the electronic supplementary material.

Supplementary material 1 (DOCX 1214 KB)
