# Supplementary material for: Genetic analysis of roots and shoots in rice seedling by association mapping
Source: Genes Genomics. 2018 Sep 21;41(1):95–105. doi: 10.1007/s13258-018-0741-x (PMC6336761; doi:10.1007/s13258-018-0741-x)
Supplement: Supplementary file 1 — Supplementary material 1 (DOCX 1214 KB) [file 13258_2018_741_MOESM1_ESM.docx]

**Supplemental files of**

**Genetic Analysis of Roots and Shoots in Rice Seedling by Association Mapping**

Zhao Yan^*1^, Jiang Cong-hui^*1^, Rashid Muhammad Abdul Rehman^*1,2^, Zhang Hong-liang^1^, Li Jinjie^1^ and Li Zi-chao^1^

^1^ Key Lab of Crop Heterosis and Utilization of Ministry of Education and Beijing Key Lab of Crop Genetic Improvement, China Agricultural University, Beijing, 100193, China.

^2^ Central Hi-Tech Lab, University of Agriculture Faisalabad, Sub-campus Burewala, Vehari, 61010, Pakistan.

* indicate the authors who contributed equally to this study

Correspondence Zichao Li, Professor, Tel: +86-010-62731414, E-mail: [lizichao@cau.edu.cn](mailto:lizichao@cau.edu.cn)

**Fig. S1** QQ-plots of general linear model (GLM) for six traits in full population. The horizontal black dashed lines in QQ-plots show thresholds at *P* = 0.05 after Bonferroni-adjusted multiple test correction.

**Fig. S2** QQ-plots of GLM for six traits in *indica*. The horizontal black dashed lines in QQ-plots show thresholds at *P* = 0.05 after Bonferroni-adjusted multiple test correction.

**Fig. S3** QQ-plots of GLM for six traits in *japonica*. The horizontal black dashed lines in QQ-plots of show thresholds at *P* = 0.05 after Bonferroni-adjusted multiple test correction.

**Fig. S4** QQ-plots of mixed linear model (MLM) for six traits in full population. The horizontal black dashed lines in QQ-plots show thresholds at *P* = 0.05 after Bonferroni-adjusted multiple test correction.

**Fig. S5** QQ-plots of MLM for six traits in *indica*. The horizontal black dashed lines in QQ-plots show thresholds at *P* = 0.05 after Bonferroni-adjusted multiple test correction.

**Fig. S6** QQ-plots of MLM for six traits in *japonica*. The horizontal black dashed lines in QQ-plots show thresholds at *P* = 0.05 after Bonferroni-adjusted multiple test correction.

**Fig. S7** Manhattan plots of GLM for six traits in full population. The horizontal black dashed lines in Manhattan plots show thresholds at *P* = 0.05 after Bonferroni-adjusted multiple test correction.

**Fig. S8** Manhattan plots of GLM for six traits in *indica*. The horizontal black dashed lines in Manhattan plots show thresholds at *P* = 0.05 after Bonferroni-adjusted multiple test correction.

**Fig. S9** Manhattan plots of GLM for six traits in *japonica*. The horizontal black dashed lines in Manhattan plots show thresholds at *P* = 0.05 after Bonferroni-adjusted multiple test correction.

**Fig. S10** Manhattan plots of MLM for six traits in full population. The horizontal black dashed lines in Manhattan plots show thresholds at *P* = 0.05 after Bonferroni-adjusted multiple test correction.

**Fig. S11** Manhattan plots of MLM for six traits in *indica*. The horizontal black dashed lines in Manhattan plots show thresholds at *P* = 0.05 after Bonferroni-adjusted multiple test correction.

**Fig. S12** Manhattan plots of MLM for six traits in *japonica*. The horizontal black dashed lines in Manhattan plots show thresholds at *P* = 0.05 after Bonferroni-adjusted multiple test correction.

**Table S1** Nutrient solution for hydroponic culture of rice

| **Element** | **Chemical** | **Concentration of mother liquor (800×)** |
| --- | --- | --- |
| N | NH_4_NO_3_ | 91.4 (g/L) |
| P | NaH_2_PQ_4_·2H_2_O | 40.3 (g/L) |
| K | K_2_SO_4_ | 71.4 (g/L) |
| Ca | CaCl_2_ | 88.6 (g/L) |
| Mg | MgSO_4_·7H_2_O | 324 (g/L)) |
| Si | Na_2_SiS_3_·9H_2_O | 18.84 (g/L) |
| Mn | MnC1_2_·4H_2_O | 1.5 (g/L) |
| Mo | (NH_4_)_6_·Mo_7_O_24_·4H_2_O | 0.074 (g/L) |
| B | H_3_BO_3_ | 0.934 (g/L) |
| Zn | ZnSO_4_·7H_2_O | 0.035 (g/L) |
| Cu | CuSO_4_·5H_2_O | 0.031 (g/L) |
| Fe | FeCl_3_·6H_2_O | 7.7 (g/L) |

**Table S2** Descriptive statistics of six seedling traits

| **Traits** | **Period** | **Range** | **Mean ± stdev** | **CV(%)** | ***h^2^*(%)** | **Skewness** | **Kurtosis** | **Correlation** |
| --- | --- | --- | --- | --- | --- | --- | --- | --- |
| RL(cm) | 1 | 8.13～18.32 | 13.71±1.70 | 12.4 | 77.63 | -0.389 | 0.66 | .716** |
|  | 2 | 8.03～19.83 | 12.67± 1.92 | 15.2 | 72.86 | 0.385 | -0.184 |  |
| RT(mm) | 1 | 0.54～1.02 | 0.77±0.08 | 10.5 | 66.23 | 0.46 | 0.416 | .607** |
|  | 2 | 0.45～0.84 | 0.65±0.07 | 10.2 | 62.95 | -0.024 | 0.332 |  |
| RW(g) | 1 | 122.50～642.25 | 386.40±109.75 | 28.4 | 84.97 | -0.109 | -0.476 | .601** |
|  | 2 | 60.10～375.47 | 198.41±51.21 | 25.8 | 73.52 | 0.412 | 0.655 |  |
| SL(cm) | 1 | 15.24～35.98 | 25.37±4.55 | 17.9 | 93.32 | 0.247 | -0.781 | .849** |
|  | 2 | 16.73～38.13 | 26.83±4.04 | 15.1 | 80.71 | 0.388 | -0.184 |  |
| SW(g) | 1 | 200.68～917.16 | 540.64±150.58 | 27.9 | 87.48 | 0.077 | -0.444 | .753** |
|  | 2 | 199.86～668.14 | 376.04±86.01 | 22.9 | 75.53 | 0.445 | 0.655 |  |
| R/S | 1 | 0.45～1.15 | 0.72±0.10 | 14.5 | 75.6 | 0.503 | 1.016 | .445** |
|  | 2 | 0.24～0.88 | 0.53±0.09 | 17.1 | 62.46 | -0.039 | 0.609 |  |

CV: coefficient of variation.

*h^2^*: broad-sense heritability.

Repeatability: Correlation between two replication for each trait

**: represent significant correlation at α = 0.01

**Table S3** Descriptive statistics of six seedling traits in *indica* and *japonica*

| Trait | Subgroup | Mean ± SD | Range | *p*-value (two-tailed t-test) |
| --- | --- | --- | --- | --- |
| SL | *jap.* | 25.89±3.63 | 17.57～35.33 | 0.65 |
|  | *ind.* | 26.13±4.36 | 16.44～36.22 |  |
| RL | *jap.* | 13.35±1.66 | 8.74～18.16 | 0.10 |
|  | *ind.* | 12.98±1.68 | 8.37～16.61 |  |
| RW | *jap.* | 272±65 | 150～442 | 0.01** |
|  | *ind.* | 304±77 | 128～484 |  |
| SW | *jap.* | 425±108 | 248～722 | 0.00** |
|  | *ind.* | 480±110 | 222～732 |  |
| RT | *jap.* | 0.73±0.07 | 0.52～0.88 | 0.00** |
|  | *ind.* | 0.69±0.06 | 0.53～0.9 |  |
| R/S | *jap.* | 0.65±0.08 | 0.46～0.87 | 0.32 |
|  | *ind.* | 0.63±0.08 | 0.43～0.92 |  |

** represent significant difference at α = 0.01.

**Table S4** QTLs related to six seedling traits by association mapping of GLM (Q) in full population, *indica* and *japonica*

| Trait | QTL | Marker | Chr. | Position (Mb) | -Log ( *P*) | | | PVE (%) | | | Previous mapping |
| --- | --- | --- | --- | --- | --- | --- | --- | --- | --- | --- | --- |
|  |  |  |  |  | Full pop. | *Ind.* | *Jap.* | Full pop. | *Ind.* | *Jap.* |  |
| RL | *qRL1-1* | RM1141 | 1 | 1.62 | 2.63 | - | - | 7.61 | - | - | 1 |
|  | *qRL1-2* | RM5302 | 1 | 4.15 | - | - | 1.81 | - | - | 14.82 | 1 |
|  | *qRL1-3* | RM7086 | 1 | 6.19 | 2.88 | - | - | 8.31 | - | - | - |
|  | *qRL1-4*^b^ | RM5496 | 1 | 8.07 | - | - | 1.7 | - | - | 13.95 | - |
|  | *qRL2-1*^bc^ | RM5300 | 2 | 33.86 | 2.11 | - | - | 6.16 | - | - | 2 |
|  | *qRL3-1* | RM251 | 3 | 0 | - | 3.24 | - | - | 13.81 | - | - |
|  | *qRL3-2*^abc^ | RM6849 | 3 | 3.27 | 3.22 | 2.05 | 1.71 | 9.24 | 8.99 | 9.86 | - |
|  | *qRL3-3*^a^ | RM6837 | 3 | 7.44 | - | 2.11 | 1.89 | - | 9.22 | 15.39 | - |
|  | *qRL3-4* | RM5944 | 3 | 8.79 | - | - | 2.52 | - | - | 19.99 | - |
|  | *qRL3-5* | RM2187 | 3 | 35.56 | - | - | 1.72 | - | - | 14.08 | - |
|  | *qRL4-1* | RM1112 | 4 | 34.2 | - | - | 1.81 | - | - | 14.76 | 3 |
|  | *qRL6-1*^bc^ | RM3183 | 6 | 12.29 | 2.09 | - | 2.72 | 6.08 | - | 21.36 | - |
|  | *qRL7-1*^c^ | RM6872 | 7 | 4.69 | 2.55 | - | 2.27 | 7.39 | - | 18.19 | - |
|  | *qRL8-1*^bc^ | RM152 | 8 | 0.68 | - | 3.1 | - | - | 13.24 | - | - |
|  | *qRL8-2*^b^ | RM126 | 8 | 5.22 | 3.37 | 2.56 | - | 9.65 | 11.08 | - | - |
|  | *qRL9-1* | RM108 | 9 | 19.01 | 2.41 | 2.79 | - | 7.01 | 12.02 | - | 6 |
|  | *qRL9-2*^b^ | TC136 | 9 | 19.08 | 3.15 | 2.68 | - | 9.04 | 11.55 | - | 6 |
|  | *qRL12-1* | RM3455 | 12 | 4.92 | 2.45 | 3.45 | - | 7.11 | 14.6 | - | - |
|  | *qRL12-2* | RM1015 | 12 | 22.41 | 2.69 | - | - | 7.77 | - | - | - |
|  | *qRL12-3*^b^ | TC19 | 12 | 25.37 | - | 2.26 | - | - | 9.85 | - | 2 |
| RT | *qRT1-1* | TC55 | 1 | 9.34 | 2.27 | - | 1.96 | 4.64 | - | 10.11 | - |
|  | *qRT1-2*^a^ | RM7075 | 1 | 15.1 | - | 2.14 | 2.1 | - | 9.38 | 14.84 | - |
|  | *qRT1-3* | RM165 | 1 | 40.1 | - | 3.58 | - | - | 12.39 | - | 3 |
|  | *qRT1-4* | OSR23 | 1 | 40.43 | - | 3.28 | - | - | 13.98 | - | 3 |
|  | *qRT1-5* | RM104 | 1 | 40.49 | - | 3.24 | - | - | 13.83 | - | 3 |
|  | *qRT2-1* | RM3188 | 2 | 3.45 | - | 2.16 | - | - | 9.45 | - | - |
|  | *qRT2-2* | RM7006 | 2 | 6.13 | - | - | 3.13 | - | - | 21.14 | 1 |
|  | *qRT2-3* | RM7009 | 2 | 32.98 | - | - | 2.31 | - | - | 16.17 | 5 |
|  | *qRT3-1* | RM3117 | 3 | 3.77 | 2.98 | 3.23 | - | 8.07 | 13.81 | - | 3 |
|  | *qRT3-2* | RM6283 | 3 | 16.78 | 3.35 | 2.94 | - | 9.03 | 12.64 | - | - |
|  | *qRT4-1* | RM3217 | 4 | 30.34 | 2.9 | 2.27 | - | 7.86 | 9.94 | - | 8 |
|  | *qRT5-1*^b^ | RM1187 | 5 | 23.13 | - | 2.14 | - | - | 9.37 | - | 2 |
|  | *qRT5-2* | RM178 | 5 | 25.08 | 3.38 | - | - | 9.11 | - | - | - |
|  | *qRT6-1* | RM225 | 6 | 3.42 | - | 2.74 | - | - | 11.86 | - | 2 |
|  | *qRT6-2* | RM5350 | 6 | 4.91 | - | - | 2.58 | - | - | 17.83 | 2 |
|  | *qRT8-1* | TC110 | 8 | 21.81 | 2.8 | - | - | 7.6 | - | - | 4 |
|  | *qRT9-1* | RM219 | 9 | 7.83 | 2.55 | 2.1 | - | 6.94 | 9.2 | - | 1 |
|  | *qRT11-1* | RM5961 | 11 | 19.22 | - | 2.22 | - | - | 9.71 | - | 3 |
|  | *qRT11-2* | RM224 | 11 | 26.8 | 3.28 | - | - | 8.84 | - | - | 1 |
|  | *qRT12-1*^b^ | RM117 | 12 | 0 | - | 2.48 | - | - | 10.8 | - | 2 |
|  | *qRT12-2*^b^ | TC19 | 12 | 25.37 | - | - | 1.94 | - | - | 13.79 | 1 |
| RW | *qRW1-1*^b^ | RM5496 | 1 | 8.07 | - | - | 1.96 | - | - | 14.43 | 1 |
|  | ***qRW1-2^c^*** | RM113 | 1 | 19.16 | - | 1.92 | - | - | 8.72 | - | 1 |
|  | ***qRW1-3*^c^** | RM5 | 1 | 23.97 | 2.7 | 3.18 | - | 7.72 | 13.98 | - | 1 |
|  | *qRW2-1* | RM109 | 2 | 0.18 | 2.52 | - | - | 7.23 | - | - | 1 |
|  | *qRW2-2* | RM106 | 2 | 25.14 | 2.4 | - | 2.37 | 6.9 | - | 17.19 | - |
|  | ***qRW2-3*^bc^** | RM5300 | 2 | 33.86 | 4.1 | 3.11 | - | 11.47 | 13.69 | - | 2 |
|  | ***qRW3-1*^bc^** | RM6849 | 3 | 3.27 | 2.67 | - | 2.63 | 7.63 | - | 14.65 | 1 |
|  | *qRW3-2* | RM3126 | 3 | 3.66 | - | - | 2.12 | - | - | 15.49 | 1 |
|  | *qRW3-3* | TC130 | 3 | 3.82 | - | 2.2 | - | - | 9.9 | - | 1 |
|  | *qRW3-4* | RM130 | 3 | 33.18 | - | 2.35 | - | - | 10.55 | - | - |
|  | *qRW4-1* | RM255 | 4 | 30.99 | - | - | 2.09 | - | - | 15.31 | - |
|  | *qRW5-1*^b^ | RM1187 | 5 | 23.13 | - | 2.87 | - | - | 12.71 | - | - |
|  | ***qRW6-1*^c^** | RM7083 | 6 | 0.45 | - | 2.31 | - | - | 10.38 | - | - |
|  | ***qRW6-2*^abc^** | RM3183 | 6 | 12.29 | 3.27 | 1.96 | 3.01 | 9.27 | 8.88 | 21.24 | - |
|  | *qRW7-1* | RM82 | 7 | 3.16 | - | - | 2.27 | - | - | 16.54 | - |
|  | *qRW8-1*^c^ | TC144 | 8 | 0.05 | 2.46 | - | - | 7.05 | - | - | - |
|  | *qRW8-2*^bc^ | RM152 | 8 | 0.68 | - | 2.2 | - | - | 9.92 | - | - |
|  | *qRW8-3*^b^ | RM126 | 8 | 5.22 | - | 2.25 | - | - | 10.13 | - | - |
|  | *qRW8-4* | RM42 | 8 | 19.96 | 3.09 | 2.36 | - | 8.78 | 10.57 | - | - |
|  | *qRW9-1*^c^ | RM3700 | 9 | 15.43 | - | - | 1.75 | - | - | 13.03 | 1 |
|  | *qRW9-2b* | TC136 | 9 | 19.08 | 2.48 | - | - | 7.12 | - | - | 1 |
|  | ***qRW10-1*^c^** | RM6144 | 10 | 15.16 | 2.63 | - | 2.25 | 7.51 | - | 16.38 | - |
|  | *qRW10-2* | RM1146 | 10 | 19.17 | - | - | 2.19 | - | - | 16.01 | - |
|  | *qRW12-1*^b^ | RM117 | 12 | 0 | - | 2.19 | - | - | 9.88 | - | - |
| SL | *qSL1-1* | RM81a | 1 | 1.93 | 3.81 | 4.04 | - | 10.95 | 17.78 | - | - |
|  | *qSL1-2* | RM5359 | 1 | 7.18 | - | - | 2.18 | - | - | 12.14 | - |
|  | *qSL1-3* | RM3627 | 1 | 10.31 | 2.72 | - | - | 7.93 | - | - | - |
|  | *qSL1-4*^bc^ | RM5 | 1 | 23.97 | 6.3 | 5.16 | - | 17.45 | 22.12 | - | - |
|  | *qSL1-5*^b^ | RM1232 | 1 | 27.63 | 3.25 | - | - | 9.41 | - | - | - |
|  | *qSL1-6^b^* | RM1095 | 1 | 30.92 | 4.28 | 4.76 | - | 12.22 | 20.6 | - | - |
|  | *qSL3-1* | RM3203 | 3 | 0.78 | - | - | 1.84 | - | - | 10.39 | - |
|  | *qSL3-2* | TC146 | 3 | 2.04 | - | - | 2.67 | - | - | 14.55 | - |
|  | *qSL4-1* | RM335 | 4 | 0.68 | 4.14 | 7.03 | - | 11.84 | 28.87 | - | - |
|  | *qSL5-1* | RM164 | 5 | 19.18 | 3.52 | 2.64 | - | 10.16 | 12.02 | - | - |
|  | *qSL6-1*^b^ | RM1163 | 6 | 4.19 | 2.65 | - | - | 7.74 | - | - | - |
|  | *qSL7-1* | RM172 | 7 | 29.56 | - | 2.92 | - | - | 13.22 | - | - |
|  | *qSL8-1*^c^ | RM152 | 8 | 0.68 | 2.84 | - | - | 8.29 | - | - | - |
|  | *qSL8-2* | RM5767 | 8 | 18.81 | - | - | 3.41 | - | - | 14.75 | - |
|  | *qSL8-3* | RM223 | 8 | 20.65 | 2.74 | 2.57 | - | 8.01 | 11.73 | - | - |
|  | *qSL8-4*^b^ | RM3262 | 8 | 22.25 | 3.04 | - | 4.71 | 8.82 | - | 23.58 | - |
|  | *qSL9-1c* | RM3700 | 9 | 15.43 | 4.4 | 4.03 | - | 12.52 | 17.75 | - | - |
|  | *qSL11-1* | KM186 | 11 | 0 | 2.67 | - | - | 6.01 | - | - | - |
|  | *qSL11-2* | RM6544 | 11 | 3.85 | 3.9 | - | - | 11.19 | - | - | - |
|  | *qSL11-3* | RM4504 | 11 | 5.47 | 5.11 | 5.32 | - | 14.39 | 22.73 | - | - |
|  | *qSL12-1* | RM235 | 12 | 26.11 | 3.25 | - | - | 9.4 | - | - | - |
|  | *qSL12-2* | RM1227 | 12 | 27.37 | - | - | 2.61 | - | - | 14.27 | - |
| SW | ***qSW1-1*^c^** | RM113 | 1 | 19.16 | 2.05 | 2.54 | - | 5.73 | 11.15 | - | - |
|  | ***qSW1-2*^bc^** | RM5 | 1 | 23.97 | 3.18 | 5.47 | - | 8.75 | 22.42 | - | - |
|  | *qSW1-3*^b^ | RM1232 | 1 | 27.63 | - | 2.01 | - | - | 8.94 | - | - |
|  | *qSW1-4*^b^ | RM1095 | 1 | 30.92 | 3.07 | 3.73 | - | 8.45 | 15.92 | - | - |
|  | *qSW2-1* | RM211 | 2 | 0 | 2.32 | - | - | 6.45 | - | - | - |
|  | ***qSW2-2*^c^** | RM5300 | 2 | 33.86 | 3.97 | 2.7 | - | 10.77 | 11.82 | - | - |
|  | *qSW3-1* | RM231 | 3 | 2.44 | 3.42 | - | 2.14 | 9.37 | - | 15.83 | - |
|  | ***qSW3-2*^c^** | RM6849 | 3 | 3.27 | - | - | 1.79 | - | - | 9.57 | - |
|  | *qSW3-3* | RM8203 | 3 | 31.19 | - | 1.92 | - | - | 8.57 | - | - |
|  | *qSW4-1* | RM5320 | 4 | 27.8 | - | - | 2.62 | - | - | 18.98 | - |
|  | *qSW5-1* | RM1089 | 5 | 5.34 | - | - | 2.06 | - | - | 15.3 | - |
|  | ***qSW6-1*^c^** | RM7083 | 6 | 0.45 | 2.23 | 2.19 | - | 6.23 | 9.71 | - | - |
|  | *qSW6-2*^b^ | RM1163 | 6 | 4.19 | 2.25 | - | - | 6.28 | - | - | - |
|  | ***qSW6-3*^c^** | RM3183 | 6 | 12.29 | 2.26 | - | 1.96 | 6.31 | - | 14.6 | - |
|  | *qSW7-1* | RM5344 | 7 | 1.9 | 2.38 | - | - | 6.62 | - | - | - |
|  | *qSW7-2*^c^ | RM6872 | 7 | 4.69 | - | - | 1.72 | - | - | 12.96 | - |
|  | *qSW8-1*^c^ | TC144 | 8 | 0.05 | 2.71 | 2.45 | - | 7.51 | 10.77 | - | - |
|  | *qSW8-2*^b^ | RM3262 | 8 | 22.25 | 3.19 | - | - | 8.77 | - | - | - |
|  | *qSW9-1* | RM3249 | 9 | 19.75 | - | - | 1.74 | - | - | 13.06 | - |
|  | ***qSW10-1*^c^** | RM6144 | 10 | 15.16 | - | - | 2.04 | - | - | 15.12 | - |
|  | *qSW11-1* | TC126 | 11 | 24.1 | - | - | 1.81 | - | - | 13.56 | - |
| R/S | *qRS1-1* | RM3234 | 1 | 7.57 | 2.36 | - | - | 6.86 | - | - | - |
|  | *qRS1-2* | RM1095 | 1 | 30.92 | - | 1.72 | - | - | 7.99 | - | - |
|  | *qRS1-3* | RM1183 | 1 | 30.97 | 2.45 | - | - | 7.13 | - | - | - |
|  | *qRS2-1* | RM106 | 2 | 25.14 | 3.49 | - | 3.26 | 9.97 | - | 24.78 | - |
|  | *qRS2-2* | RM5460 | 2 | 33.77 | 2.18 | - | 2.86 | 6.37 | - | 22.07 | - |
|  | *qRS3-1* | RM2614 | 3 | 27.23 | 2.42 | - | 2.54 | 7.02 | - | 19.91 | - |
|  | *qRS4-1* | RM255 | 4 | 30.99 | 2.24 | 1.69 | - | 6.53 | 7.85 | - | - |
|  | *qRS5-1* | RM5361 | 5 | 0.49 | 2.33 | - | - | 6.78 | - | - | - |
|  | *qRS5-2* | RM1024 | 5 | 1.17 | 2.51 | - | 2.45 | 7.3 | - | 19.24 | - |
|  | *qRS5-3* | RM1187 | 5 | 23.13 | - | 2.31 | - | - | 10.6 | - | - |
|  | *qRS6-1* | TC04 | 6 | 28.2 | - | - | 2.67 | - | - | 20.8 | - |
|  | *qRS6-2* | Rm103 | 6 | 30.89 | - | - | 2.48 | - | - | 19.5 | - |
|  | *qRS7-1* | RM82 | 7 | 3.16 | - | - | 2.18 | - | - | 17.36 | - |
|  | *qRS8-1* | RM1235 | 8 | 1.2 | - | 1.7 | - | - | 7.9 | - | - |
|  | *qRS8-2* | RM42 | 8 | 19.96 | 2.38 | - | - | 6.92 | - | - | - |
|  | *qRS8-3* | RM6845 | 8 | 27.56 | - | - | 2.21 | - | - | 17.58 | - |
|  | *qRS9-1* | RM296 | 9 | 10.73 | 2.8 | - | - | 8.1 | - | - | - |
|  | *qRS9-2* | RM7175 | 9 | 16.87 | - | 2.09 | - | - | 9.63 | - | - |
|  | *qRS11-1* | RM5349 | 11 | 18.99 | 2.77 | - | - | 8 | - | - | - |
|  | *qRS11-2* | RM5961 | 11 | 19.22 | 1.92 | - | - | 5.61 | - | - | - |

^a^ QTL re-identified in *indica* and *japonica*

^b^ QTL related to several root traits or several shoot traits

^c^ QTL related to several root and shoot traits

Note: The BOLD QTL-name represent the pleiotropic QTLs, associated with root weight and shoot weight.

Last column “Previous mapping” showed the number of repeated-identification by linkage mapping ([Courtois et al. 2009](#_ENREF_3)).

**Table S5** QTLs related to six seedling traits by association mappings of GLM (Q) and MLM (Q+K) in full population, *indica* and *japonica*

| Trait | QTL | Marker | Chr | Position (Mb) | -Log (*P*) | | | PVE (%) | | | Previous mapping |
| --- | --- | --- | --- | --- | --- | --- | --- | --- | --- | --- | --- |
|  |  |  |  |  | Full pop. | *Ind.* | *Jap.* | Full pop. | *Ind.* | *Jap.* |  |
| RL | *qRL1-2* | RM5302 | 1 | 4.15 | 1.83 |  |  | 7.01 |  |  | 1 |
|  | *qRL3-3* | RM6837 | 3 | 7.44 |  | 1.96 |  |  | 14.13 |  | - |
|  | *qRL7-1* | RM6872 | 7 | 4.69 | 2.59 |  |  | 11.11 |  |  | - |
|  | *qRL12-2* | RM1015 | 12 | 22.41 | 1.75 |  |  | 18.64 |  |  | - |
| RT | *qRT1-3* | Rm165 | 1 | 40.1 |  | 2.02 |  |  | 7 |  | 3 |
|  | *qRT1-5* | RM104 | 1 | 40.49 |  | 1.63 |  |  | 11.92 |  | 3 |
|  | *qRT2-2* | RM7006 | 2 | 6.13 |  |  | 2 |  |  | 21.87 | 1 |
|  | *qRT5-1*^b^ | RM1187 | 5 | 23.13 |  | 2.03 |  |  | 14.32 |  | 2 |
|  | *qRT11-1* | RM5961 | 11 | 19.22 | 2.29 |  |  | 13.92 |  |  | 3 |
| RW | *qRW2-2* | RM106 | 2 | 25.14 | 2.05 |  | 1.62 | 7.84 |  | 14.46 | - |
|  | *qRW3-3* | TC130 | 3 | 3.82 |  | 2.25 |  |  | 16.09 |  | 1 |
|  | *qRW3-4* | RM130 | 3 | 33.18 |  | 1.84 |  |  | 13.5 |  | - |
|  | *qRW5-1*^b^ | RM1187 | 5 | 23.13 |  | 1.72 |  |  | 12.8 |  | - |
|  | *qRW6-1* | RM7083 | 6 | 0.45 | 1.62 |  |  | 6.33 |  |  | - |
|  | ***qRW6-2*** | RM3183 | 6 | 12.29 | 1.84 |  | 1.53 | 11.92 |  | 21.13 | - |
|  | *qRW10-1* | RM6144 | 10 | 15.16 | 1.9 |  | 1.68 | 7.31 |  | 19.17 | - |
|  | *qRW10-2* | RM1146 | 10 | 19.17 | 2.06 |  | 1.63 | 14.05 |  | 32.9 | - |
| SL | *qSL8-2* | RM5767 | 8 | 18.81 |  |  | 2 |  |  | 22.04 | - |
|  | *qSL8-4*^b^ | RM3262 | 8 | 22.25 |  |  | 1.86 |  |  | 26.87 | - |
|  | *qSL11-1* | KM186 | 11 | 0 | 2.62 | 1.72 |  | 9.85 | 10.7 |  | - |
|  | *qSL11-2* | RM6544 | 11 | 3.85 | 3.01 |  |  | 14.25 |  |  | - |
| SW | *qSW1-4* | RM1095 | 1 | 30.92 | 1.93 | 2.12 |  | 14.52 | 25.2 |  | - |
|  | *qSW4-1* | RM5320 | 4 | 27.8 | 2.97 |  | 2.39 | 15.47 |  | 22.39 | - |
|  | ***qSW6-3*** | RM3183 | 6 | 12.29 |  | 1.58 |  |  | 15.61 |  | - |
|  | *qSW8-1* | TC144 | 8 | 0.05 |  | 1.62 |  |  | 15.93 |  | - |
|  | *qSW8-2*^b^ | RM3262 | 8 | 22.25 | 2.44 | 1.57 |  | 14.54 | 11.89 |  | - |
|  | *qSW11-1* | TC126 | 11 | 24.1 |  |  | 1.57 |  |  | 40.61 | - |
| R/S | *qRS2-1* | RM106 | 2 | 25.14 | 1.95 |  |  | 7.49 |  |  |  |
|  | *qRS4-1* | rm255 | 4 | 30.99 | 1.97 | 2.02 |  | 19.03 | 26.26 |  |  |
|  | *qRS11-2* | RM5961 | 11 | 19.22 | 1.68 | 1.7 |  | 11.21 | 16.43 |  |  |

^b^ QTL related to several root traits or several shoot traits

^c^ QTL related to several root and shoot traits

Note: The BOLD QTL-name represent the pleiotropic QTLs, associated with root weight and shoot weight.

Last column “Previous mapping” showed the number of repeated-identification by linkage mapping ([Courtois et al. 2009](#_ENREF_3)).

**Table S6** Phenotypic statistics of nine elite varieties with positive genotypes of four pleiotropic QTLs in *indica*

| Variety | Positive genotypes | | | | Root weight  (mg) | Shoot weight  (mg) | Root length  (cm) | Root thickness  (mm) | Shoot length  (cm) |
| --- | --- | --- | --- | --- | --- | --- | --- | --- | --- |
|  | RM113 | RM5300 | RM7083 | RM5 |  |  |  |  |  |
| Z161 | 117:117 | 104:104 | 113:113 | 104:104 | 311 | 520 | 13.44 | 0.81 | 29.02 |
| zw46 | 117:117 | 104:104 | 113:113 | 108:108 | 316 | 498 | 11.68 | 0.67 | 23.01 |
| Z27 | 117:117 | 104:104 | 113:113 | 104:104 | 325 | 518 | 13.56 | 0.65 | 30.62 |
| Z152 | 117:117 | 104:104 | 113:113 | 104:104 | 337 | 514 | 12.52 | 0.77 | 22.97 |
| Z41 | 117:117 | 104:104 | 113:113 | 104:104 | 347 | 678 | 14.34 | 0.69 | 32.68 |
| Z117 | 117:117 | 104:104 | 113:113 | 104:104 | 350 | 605 | 14.43 | 0.67 | 29.6 |
| Z54 | 117:117 | 104:104 | 113:113 | 108:108 | 365 | 619 | 15.76 | 0.9 | 26.06 |
| Z8 | 117:117 | 104:104 | 113:113 | 108:108 | 400 | 650 | 14.44 | 0.71 | 29.66 |
| zw19 | 117:117 | 104:104 | 113:113 | 108:108 | 432 | 725 | 15.32 | 0.69 | 35.54 |

**Table S7** Phenotypic statistics of 20 elite varieties with positive genotypes of two pleiotropic QTLs in *japonica*

| Variety | Positive genotypes | | Root weight  (mg) | Shoot weight  (mg) | Root length  (cm) | Root thickness  (mm) | Shoot length  (cm) |
| --- | --- | --- | --- | --- | --- | --- | --- |
|  | RM3183 | RM6144 |  |  |  |  |  |
| zw61 | 102:102 | 118:118 | 289 | 339 | 13.97 | 0.7 | 21.67 |
| Z1 | 102:102 | 118:118 | 290 | 469 | 14.64 | 0.68 | 28.55 |
| zw88 | 102:102 | 118:118 | 295 | 559 | 14.04 | 0.75 | 25.75 |
| zw32 | 102:102 | 118:118 | 297 | 343 | 14.07 | 0.74 | 26.54 |
| Z177 | 102:102 | 118:118 | 312 | 474 | 15.72 | 0.72 | 22.16 |
| Z55 | 102:102 | 118:118 | 315 | 466 | 14.3 | 0.88 | 27.09 |
| Z141 | 102:102 | 118:118 | 328 | 574 | 12.39 | 0.75 | 30.94 |
| Z19 | 102:102 | 118:118 | 329 | 427 | 13.11 | 0.69 | 27.43 |
| zw58 | 102:102 | 118:118 | 330 | 577 | 15.6 | 0.88 | 31.07 |
| Z14 | 102:102 | 118:118 | 335 | 426 | 11.84 | 0.72 | 25.95 |
| zw55 | 102:102 | 118:118 | 336 | 550 | 13.92 | 0.83 | 33.83 |
| Z153 | 102:102 | 118:118 | 347 | 538 | 12.56 | 0.74 | 23.59 |
| Z87 | 102:102 | 118:118 | 349 | 487 | 14.18 | 0.74 | 32.25 |
| zw85 | 102:102 | 118:118 | 351 | 716 | 14.19 | 0.79 | 35.33 |
| Z94 | 102:102 | 118:118 | 361 | 480 | 17.66 | 0.81 | 26.74 |
| Z91 | 102:102 | 118:118 | 367 | 434 | 12.81 | 0.82 | 25.52 |
| zw86 | 102:102 | 118:118 | 373 | 686 | 14.5 | 0.75 | 31.17 |
| Z22 | 102:102 | 118:118 | 391 | 626 | 14.08 | 0.87 | 29.16 |
| zw54 | 102:102 | 118:118 | 397 | 636 | 13.54 | 0.73 | 29.97 |
| Z17 | 102:102 | 118:118 | 398 | 537 | 13.3 | 0.75 | 23 |
